# Characteristics of the Salivary Microbiota in Patients With Various Digestive Tract Cancers

**DOI:** 10.3389/fmicb.2019.01780

**Published:** 2019-08-02

**Authors:** Shinya Kageyama, Toru Takeshita, Kenji Takeuchi, Mikari Asakawa, Rie Matsumi, Michiko Furuta, Yukie Shibata, Kiyoshi Nagai, Masahiko Ikebe, Masaru Morita, Muneyuki Masuda, Yasushi Toh, Yutaka Kiyohara, Toshiharu Ninomiya, Yoshihisa Yamashita

**Affiliations:** ^1^Section of Preventive and Public Health Dentistry, Division of Oral Health, Growth and Development, Faculty of Dental Science, Kyushu University, Fukuoka, Japan; ^2^OBT Research Center, Faculty of Dental Science, Kyushu University, Fukuoka, Japan; ^3^Department of Oral and Maxillofacial Surgery, National Hospital Organization Kyushu Cancer Center, Fukuoka, Japan; ^4^Department of Gastroenterological Surgery, National Hospital Organization Kyushu Cancer Center, Fukuoka, Japan; ^5^Department of Head and Neck Surgery, National Hospital Organization Kyushu Cancer Center, Fukuoka, Japan; ^6^Hisayama Research Institute for Lifestyle Diseases, Fukuoka, Japan; ^7^Department of Epidemiology and Public Health, Graduate School of Medical Sciences, Kyushu University, Fukuoka, Japan

**Keywords:** saliva, oral microbiota, digestive tract cancer, tongue cancer, pharyngeal cancer, esophageal cancer, gastric cancer, colorectal cancer

## Abstract

The salivary microbiota is constantly swallowed and delivered to the digestive tract. These bacteria may be associated with gastrointestinal diseases. This case-control study examined the salivary microbiota in patients with digestive tract cancer (DTC) and evaluated their differential distribution based on the cancer sites. We collected saliva samples from 59 patients with cancer in any part of the digestive tract (tongue/pharynx, esophagus, stomach, and large intestine) and from 118 age- and sex-matched control subjects. There was no significant difference in periodontal status between DTC patients and control subjects (*P* = 0.72). We examined the bacterial diversity and composition in saliva by 16S ribosomal RNA gene sequencing. Salivary bacterial diversity in DTC patients was significantly higher than that in control subjects [number of operational taxonomic units (OTUs), *P* = 0.02; Shannon index, *P* < 0.01; Chao1, *P* = 0.04]. Eleven differentially abundant OTUs in DTC patients were identified using the linear discriminant analysis effect size (LEfSe) method. Based on the cancer sites, the diversity of salivary bacteria was especially higher in tongue/pharyngeal or esophageal cancer patients than in control subjects. Among the 11 differentially abundant OTUs in DTC patients, an OTU corresponding to *Porphyromonas gingivalis* was more abundant in the saliva of all groups of DTC patients compared to that in control subjects, and an OTU corresponding to *Corynebacterium* species was more abundant in all groups other than gastric cancer patients (*P* < 0.01). In addition, the relative abundances of OTUs corresponding to *Fusobacterium nucleatum*, *Streptococcus parasanguinis* II, and *Neisseria* species were significantly higher in tongue/pharyngeal cancer patients compared to their abundances in control subjects (*P* < 0.01). The relative abundance of an OTU corresponding to the *Neisseria* species was also significantly higher in gastric cancer patients and that of an OTU corresponding to *Actinomyces odontolyticus* was significantly higher in colorectal cancer patients (*P* < 0.01). These results suggest that the salivary microbiota might be associated with various digestive tract cancers.

## Introduction

The digestive tract comprises the oral cavity, pharynx, esophagus, stomach, and intestine. It has an indispensable role in the digestion and absorption of nutrients in the human body. Digestive tract cancer (DTC) is a malignant disease and is one of the most common cancers worldwide. In Japan, approximately 300,000 individuals are diagnosed with DTC annually (Hori et al., 2015). Among Japanese females, colorectal and gastric cancers are the first and fourth leading causes of cancer-related deaths, respectively, while among Japanese males they are the third and second leading causes, respectively (Ministry of Health, Labour and Welfare, 2018). Numerous epidemiological studies have demonstrated that smoking, alcohol intake, obesity, underweight, and low consumption of vegetables and fruits are lifestyle factors associated with DTC (Kabat et al., 1994; Bosetti et al., 2000; Engel et al., 2003; Huang et al., 2003; Ferrari et al., 2007; Freedman et al., 2007; Paskett et al., 2007; Botteri et al., 2008; Leung et al., 2008; Varela-Lema et al., 2010; Radoï et al., 2015; Shaukat et al., 2017). In addition, several studies have suggested the involvement of bacteria in several types of cancers, especially in organs continuously exposed to microbes, by the induction of chronic and persistent inflammation or the downregulation of host immunity (Allavena et al., 2008; Dalton-Griffin and Kellam, 2009; Tjalsma et al., 2012; Mima et al., 2015).

In the oral cavity, innumerable bacteria form a complex and stable bacterial community, which may play an important role in the oral and systemic diseases (Socransky et al., 1998; Pérez-Chaparro et al., 2014; Kholy et al., 2015; Mathews et al., 2016; Takeshita et al., 2016; Zaura et al., 2017; Asakawa et al., 2018; Kageyama et al., 2018). Considering that the oral microbiota is constantly swallowed along with saliva and delivered to the digestive tract, it is reasonable to consider that the oral microbiota is a possible risk factor for DTC, even in cancer sites that are distant from the oral cavity. In fact, *Fusobacterium nucleatum*, a periodontal disease pathogen, that usually inhabits the oral cavity is frequently detected and is abundant in colorectal carcinoma tissue (Mima et al., 2015; Nosho et al., 2016). Moreover, it is assumed that the causal link between oral microbiota and DTC differs by organs, because each digestive tract organ has a specific environment and an indigenous bacterial community. Evaluation of the characteristics of salivary microbiota in DTC patients according to the cancer sites may help in the elucidation of the microbial etiology of DTC, and aid in prediction or diagnosis of DTC.

Although previous studies have examined the association between the oral microbiota and DTC, interpretation of differences among these studies is not easy because the background of participants, such as race and food-related culture, differed in the studies (Peters et al., 2017; Zhao et al., 2017; Flemer et al., 2018; Yang et al., 2018). Additionally, although earlier studies suggested that several periodontal pathogens play a role in the development of DTC, no study has attempted to evaluate the relationship between oral microbiota and DTC accounting for the oral health condition, especially periodontal status, which is strongly related to bacterial diversity and composition in the oral cavity (Kageyama et al., 2017).

In this study, we examined the salivary microbiota collected from various DTC patients. We compared the bacterial diversity and composition in DTC patients with those from age- and sex-matched control subjects, using the 16S ribosomal RNA (16S rRNA) gene amplicon deep sequencing. Additionally, we accounted for the oral health condition of the subjects. The aim of this study was to compare the characteristics of the salivary microbiota in DTC patients and control subjects, and to evaluate their differential distribution by the cancer sites.

## Materials and Methods

### Study Subjects

Study subjects in this study were patients with cancer who visited the National Kyushu Cancer Center, Fukuoka, Japan. We enrolled 71 Japanese patients at their first visit for preoperative oral care prior to cancer treatment from October 2015 to February 2017. We conducted a dental examination and collected saliva samples from these patients. Patients who used antibiotics within a month preceding sampling or who had already received cancer treatment were not recruited. After excluding 12 patients who had missing clinical data (*n* = 3) and whose cancer was not located in the digestive tract (*n* = 9), 59 patients were finally included in the analysis. For control subjects, we used saliva samples and clinical data of community-dwelling people who participated in the Hisayama study, a population-based prospective study performed in the town of Hisayama (a suburban rural area of Fukuoka city in southern Japan) (Hata et al., 2013). A part of the Hisayama study conducted in 2012 comprised dental examination and saliva sampling for residents ≥39 years of age. Of the 2,654 participants who received dental examination (56.4% of the total population in this age group), 2,111 underwent saliva sampling. We randomly sampled 118 age- and sex-matched control subjects (ratio 1:2, age difference within 1 year) from 1,913 participants after excluding those with missing periodontal examination data (*n* = 2), and self-reported medical history for cancer (*n* = 196). Written informed consent was obtained from all participants. The ethics committee of Kyushu University approved this study and the informed consent procedure (Approval Number 27–37).

### Dental Examination and Sample Collection

The dental examination and sample collection in patients and control subjects were conducted according to a previously described protocol (Takeshita et al., 2016). In brief, the periodontal condition was evaluated by measuring the periodontal pocket depth (PPD) and by bleeding on probing (BOP) at two sites for all teeth (mesio- and mid-buccal sites) based on the NHANES III method. The oral hygiene status was assessed using the dental plaque score according to the Silness and Löe (1964) plaque index. Following the dental examination, the subjects chewed gum for 2 min; the stimulated saliva was collected in sterile plastic tubes. The samples were stored at −80°C until further analysis.

### 16S rRNA Gene Amplicon Sequencing of Saliva

DNA was extracted from each sample obtained from DTC patients using the bead-beating method (Yamanaka et al., 2012). The V1–V2 regions of 16S rRNA gene were amplified using the following primers: 8F (5′-AGA GTT TGA TYM TGG CTC AG-3′) with the Ion Torrent adapter A and the sample-specific 8-base index tag sequence, and 338R (5′-TGC TGC CTC CCG TAG GAG T-3′) with the Ion Torrent trP1 adapter sequence. PCR amplification, purification, and quantification of each PCR amplicon was performed as previously described (Takeshita et al., 2016). The purified PCR amplicons were pooled, and gel-purification was performed using Wizard SV Gel and PCR Clean-Up System (Promega, WI, United States). The DNA concentration was determined using a KAPA Library Quantification Kit (KAPA Biosystems, MA, United States) and the DNA was diluted for emulsion PCR. Emulsion PCR and enrichment of template-positive particles were performed using Ion PGM Hi-Q View OT2 Kit (Thermo Fisher Scientific, MA, United States) in the Ion One Touch 2 system (Thermo Fisher Scientific). The sequencing was performed on the Ion PGM (Thermo Fisher Scientific) using Ion PGM Hi-Q view Sequencing kit. DNA extraction from samples of control subjects, followed by PCR amplification, and purification were performed as described above. The purified PCR amplicons were pooled (up to 192 amplicons per pool) and sequencing was performed in 14 runs using Ion PGM Template OT2 400 Kit (Thermo Fisher Scientific) and Ion PGM Hi-Q Sequencing kit (Thermo Fisher Scientific).

### Data Analysis and Taxonomy Assignment

We completed the quality filtering of raw sequence reads using a script written in R (version 3.5.1). The reads were excluded from the analysis when they exhibited ≤200 bases, had an average quality score ≤25, did not include the correct forward primer sequence, did not include the correct reverse primer sequence (one mismatch was allowed), or had a homopolymer run >6 nucleotides. The quality-checked reads were demultiplexed by examining the 8-base tag sequence, and then forward and reverse primer sequences were trimmed. The quality-checked reads derived from 177 subjects of this study were extracted and used for further analysis. Operational taxonomic units (OTUs) were constructed by clustering quality-checked reads, excluding singleton reads, with a minimum pairwise identity of 97% using UPARSE (Edgar, 2013) as described previously (Takeshita et al., 2016). All quality-checked reads were mapped to each OTU with ≥97% identity using UPARSE (Takeshita et al., 2016). Chimeras were identified using ChimeraSlayer and removed from analysis (Haas et al., 2011). The taxonomy of representative sequences was determined using BLAST against 889 oral bacterial 16S rRNA gene sequences (HOMD 16S rRNA RefSeq version 14.51) in the Human Oral Microbiome Database (Chen et al., 2010). Nearest-neighbor species with ≥98.5% identity were selected as candidates for each representative OTU. The taxonomy of sequences without hits was further determined using an RDP classifier with a minimum support threshold of 80% (Wang et al., 2007). The number of OTUs were calculated following rarefaction to 5,000 reads per sample using the vegan package of R and were used as an index of bacterial diversity in this study. The sequence data have been deposited in DDBJ Sequence Read Archive under accession number DRA008522.

### Statistical Analyses

We compared the clinical and bacterial characteristics of patients and control subjects. Continuous variables were compared using Mann–Whitney *U*-test, and nominal or ordinal variables were compared using Fisher’s exact test. We also evaluated the characteristics of patients by their cancer site, and each characteristic was respectively compared to that of healthy control subjects using Mann–Whitney *U*-test and Fisher’s exact test. The UniFrac metric was used to determine the dissimilarity between any pair of bacterial compositions (Lozupone and Knight, 2005). Principal-coordinate analysis (PCoA) was performed based on the weighted and unweighted UniFrac distances using the cmdscale function in the stats package of R. The dissimilarity between patients and control subjects was evaluated using the analysis of similarities (ANOSIM) with 999 permutations based on weighted and unweighted UniFrac distances. The detection of discriminant bacterial species was performed using the linear discriminant analysis effect size (LEfSe) method (Segata et al., 2011). The linear discriminant analysis score (LDA score) indicated the effect size of each OTU and we defined OTUs with an LDA score >3.0 as differentially abundant OTUs.

## Results

### Characteristics of DTC Patients and Salivary Microbiota Sequence

We examined 59 DTC patients (42 men and 17 women, 38–84 years of age) and 118 age- and sex-matched control subjects. The detailed characteristics of DTC patients and control subjects are presented in Table 1. Body mass index (BMI) of DTC patients was significantly lower and the percentage of teeth with BOP was significantly higher than those in control subjects (*P* < 0.01). There were no statistical differences between DTC patients and control subjects in the other lifestyle habits and oral conditions. We analyzed a total of 177 stimulated saliva samples by 16S rRNA gene amplicon analysis, and finally obtained 2,566,571 high-quality reads (14,500 ± 5,654 reads per sample) to determine their bacterial diversity and composition.

**TABLE 1:** The characteristics of digestive tract cancer patients and control subjects.

	**Digestive tract cancer (*n* = 59)**	**Control subjects (*n* = 118)**	***P*-value**
Age, mean ± SD	66.4±10.2	66.4±10.3	0.985
Male, n (%)	42 (71.2)	84 (71.2)	1
BMI, mean ± SD	21.7±3.1	23.5±3.5	<0.01
Current smoking, n (%)	14 (24.1)	21 (17.8)	0.323
Current drinking, n (%)	32 (56.1)	70 (59.3)	0.745
Number of teeth, mean ± SD	20.8±9.3	23.3±7.0	0.148
Number of decayed teeth, mean ± SD	1.1±2.3	0.8±1.5	0.502
PPD (mm), mean ± SD	1.9±0.6	1.8±0.6	0.715
Teeth with BOP (%), mean ± SD	31.8±25.0	15.7±19.3	<0.01
Mean plaque index, mean ± SD	0.7±0.8	0.7±0.6	0.141

*SD, standard deviation; BMI, body mass index; PPD, periodontal pocket depth; BOP, bleeding on probing. Significant differences were determined using the Mann–Whitney *U*-test and the Fisher’s exact test. Fifteen individuals with ≤8 teeth were excluded in PPD, percentage of teeth with BOP, and mean plaque index.*

### Bacterial Diversity and Composition in DTC Patients and Control Subjects

**FIGURE 1:**
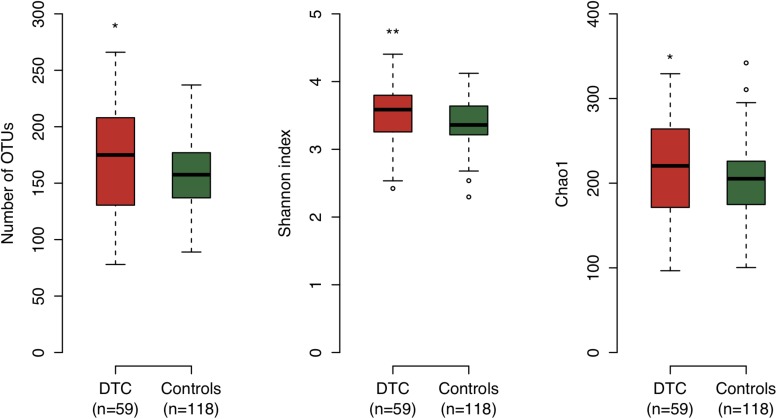
Bacterial diversity of saliva in digestive tract cancer (DTC) patients and control subjects. Boxplots show the number of operational taxonomic units (OTUs), Shannon index, and Chao1 in patients and control subjects. Significant differences were determined using the Mann–Whitney *U*-test. ^*^P < 0.05 and ^∗∗^*P* < 0.01.

**FIGURE 2:**
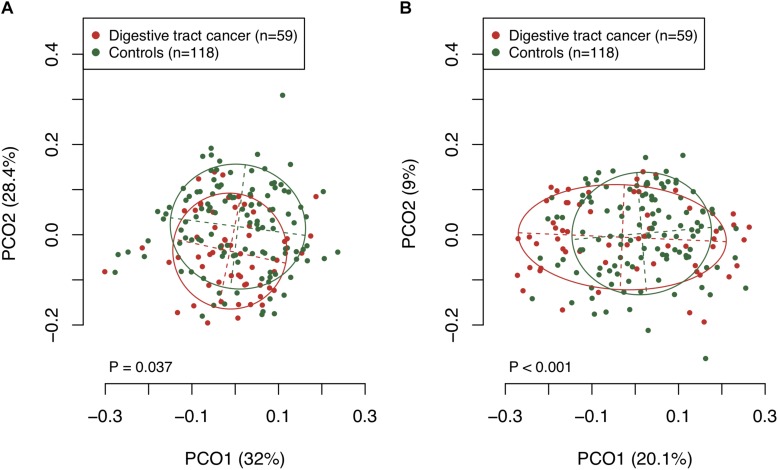
Principal coordinate analysis based on **(A)** weighted and **(B)** unweighted UniFrac distances. The bacterial composition of patients and control subjects are depicted using different colors. These two components explained the 60.4% **(A)** and 29.1% **(B)** variances. The intersection of the broken lines indicates the center of gravity for each community type. The ellipse covers 67% of the samples belonging to each community type. The *P*-value was calculated by the analysis of similarities (ANOSIM).

**FIGURE 3:**
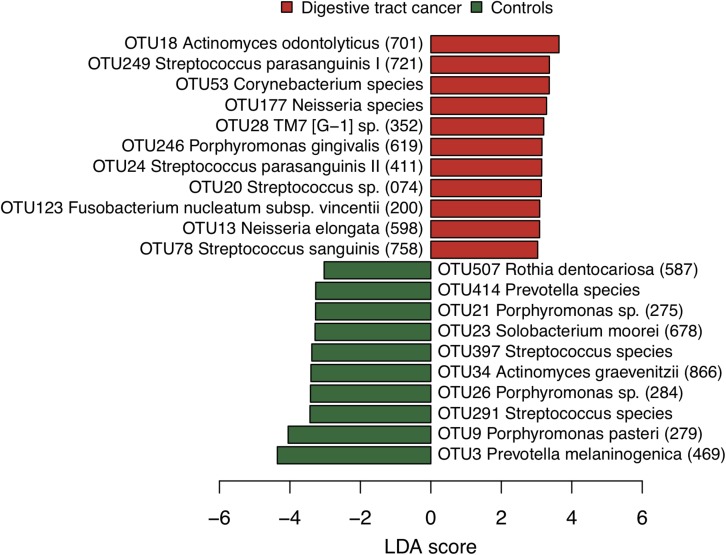
Bacterial species corresponding to the differentially abundant operational taxonomic units (OTUs) between patients and control subjects. Bar plots show linear discriminant analysis (LDA) scores of each OTU. The LDA score indicates the effect size of each OTU and OTUs with an LDA score >3.0 are shown. The differentially abundant OTUs in patients and control subjects are depicted using different colors. Oral taxon identifications are in parentheses following bacterial names.

We examined the bacterial diversity of saliva in DTC patients and control subjects to evaluate the overall salivary microbiota. The saliva of DTC patients exhibited significantly higher bacterial diversity than that of control subjects (number of OTUs, *P* = 0.02; Shannon index, *P* < 0.01; Chao1, *P* = 0.04) (Figure 1). We also evaluated the similarity of bacterial composition in saliva of patients and control subjects. Figure 2 presents a PCoA plot based on the UniFrac distances and Supplementary Figure S1 presents bar plots of dominant genera. Although there was a significant difference between DTC patients and control subjects in the overall bacterial composition of saliva according to ANOSIM (weighted, *P* = 0.037; unweighted, *P* < 0.001), a distinct difference was not observed. We also identified discriminant OTUs in salivary microbiota of DTC patients and control subjects using the LEfSe approach to evaluate the detailed difference in bacterial composition of saliva. The analysis revealed 11 OTUs including OTUs corresponding to *Actinomyces odontolyticus* HOT-701 [Human Oral Taxon (HOT) numbers are unique identification numbers in HOMD], *Streptococcus parasanguinis* I HOT-721, and *Corynebacterium* species that were differentially abundant in DTC patients compared to control subjects (Figure 3). Conversely, OTUs corresponding to *Prevotella melaninogenica* HOT-469, *Porphyromonas pasteri* HOT-279, and *Streptococcus* species were differentially abundant in control subjects. These results revealed the presence of specific bacteria in the saliva of DTC patients and control subjects.

### Characteristics of DTC Patients by the Cancer Sites

We compared the characteristics of each patient group based on their cancer sites to control subjects. The detailed characteristics of each patient group are shown in Table 2. The cancer in patients was located in the tongue/pharynx (*n* = 13), esophagus (*n* = 12), stomach (*n* = 10), and rectum/colon (*n* = 24). BMI of tongue/pharyngeal, esophageal, or gastric cancer patients were significantly lower than that of control subjects (*P* < 0.01), while there was no significant difference in BMI between colorectal cancer patients and control subjects. The proportion of current smokers in tongue/pharyngeal cancer patients was 58.3%, which was significantly higher than that in control subjects (*P* < 0.01). Concerning oral conditions, tongue/pharyngeal cancer patients demonstrated more decayed teeth, while gastric cancer patients demonstrated fewer decayed teeth compared to control subjects. Gingival inflammatory condition based on BOP in tongue/pharyngeal, esophageal, or colorectal cancer patients was more severe than in the control subjects and oral hygiene status based on plaque index was better in gastric or colorectal cancer patients. There was no difference in the periodontal condition (based on PPD) between the patient groups and control subjects.

**TABLE 2:** The characteristics of digestive tract cancer patients by the cancer sites.

	**Tongue/pharyngeal (*n* = 13)**	**Esophageal (*n* = 12)**	**Gastric (*n* = 10)**	**Colorectal (*n* = 24)**	**Control subjects (*n* = 118)**
Age, mean ± SD	62.5±10.6	68.4±7.8	70.5±9.8	65.9±10.9	66.4±10.3
Male, n (%)	12 (92.3)	8 (66.7)	6 (60)	16 (66.7)	84 (71.2)
BMI, mean ± SD	20.5±2.7^**^	20.8±3.2^**^	20.8±2.1^**^	23±3.2	23.5±3.5
Current smoking, n (%)	7 (58.3)^∗∗^	3 (25)	1 (10)	3 (12.5)	21 (17.8)
Current drinking, n (%)	9 (75)	5 (45.5)	4 (40)	14 (58.3)	70 (59.3)
Number of teeth, mean ± SD	19.8±9.9	19.2±9.9	20.9±9.5	22.1±8.9	23.3±7.0
Number of decayed teeth, mean ± SD	3.0±3.8^**^	1.2±2.0	0±0^*^	0.5±1.2	0.8±1.5
PPD (mm), mean ± SD	1.8±0.4	2.1±0.6	1.7±0.7	1.8±0.7	1.8±0.6
Teeth with BOP (%), mean ± SD	37.1±25.5^**^	40.2±27.3^**^	23.3±27.2	28.3±22.3^**^	15.7±19.3
Mean plaque index, mean ± SD	1.1±1.1	1.2±0.9	0.3±0.3^*^	0.4±0.4^*^	0.7±0.6

SD, standard deviation; BMI, body mass index; PPD, periodontal pocket depth; BOP, bleeding on probing. Significant differences were respectively determined between each patient group and control subjects using the Mann–Whitney *U*-test and the Fisher’s exact test. ^*^P < 0.05 and ^∗∗^*P* < 0.01. Fifteen individuals with ≤8 teeth were excluded in PPD, percentage of teeth with BOP, and mean plaque index.

### Bacterial Diversity and Composition in DTC Patients by the Cancer Sites

**FIGURE 4:**
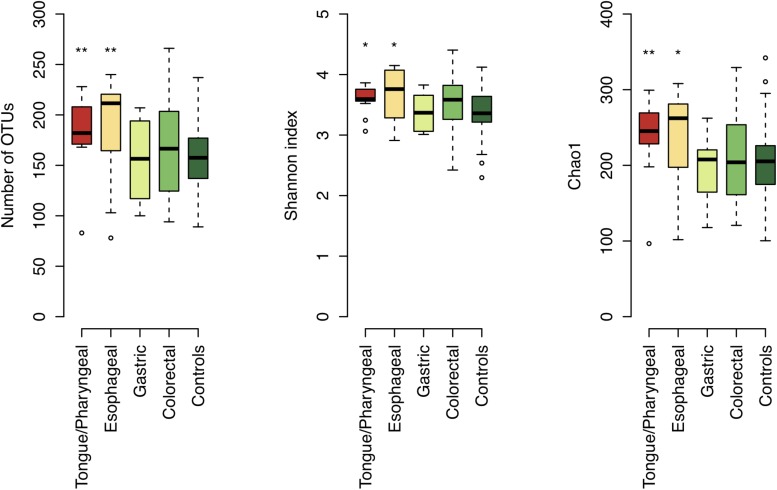
Bacterial diversity of saliva in each patient group and control subjects. Boxplots show the number of operational taxonomic units (OTUs), Shannon index, and Chao1 in each patient group and control subjects. Significant differences are respectively, determined between each patient group and control subjects using the Mann–Whitney *U*-test. ^*^P < 0.05 and ^∗∗^*P* < 0.01.

We examined the bacterial diversity of salivary microbiota in each patient group. Saliva from tongue/pharyngeal and esophageal cancer patients demonstrated significantly higher bacterial diversity compared to that of control subjects (number of OTUs, both *P* < 0.01; Shannon index, both *P* = 0.02; Chao1, *P* < 0.01 and *P* = 0.02, respectively) (Figure 4). In contrast, there was no significant difference in bacterial diversity between gastric and colorectal cancer patients and control subjects.

**FIGURE 5:**
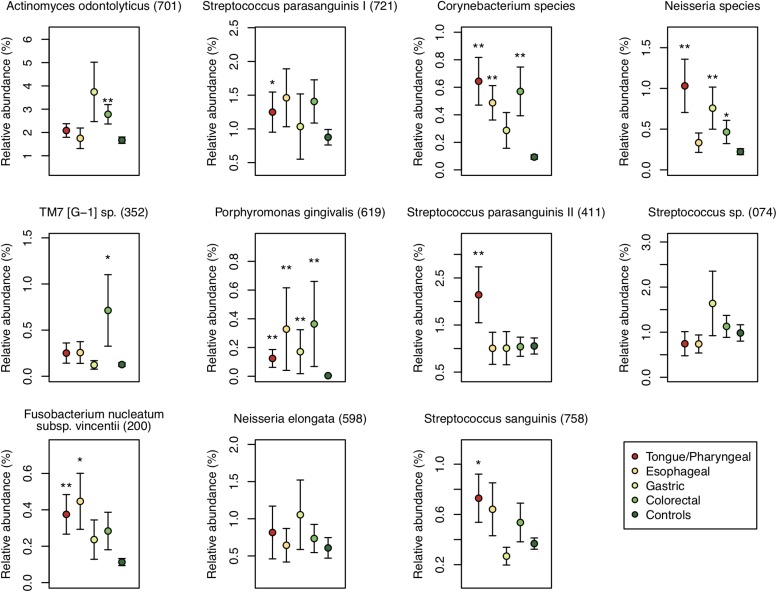
Relative abundances of the 11 differentially abundant operational taxonomic units (OTUs) in each patient group and control subjects. Each plot shows average of the relative abundance of 11 differentially abundant OTUs of patients with digestive tract cancer in each patient group, and error bar shows standard error. Each group is depicted using different colors. Oral taxon identifications are in parentheses following bacterial names. Significant differences are respectively determined between each patient group and control subjects using the Mann–Whitney *U*-test. ^*^P < 0.05 and ^∗∗^*P* < 0.01.

We also examined the relative abundance of the 11 differentially abundant OTUs in each patient group to identify the association between these OTUs and each DTC. The relative abundance of an OTU corresponding to *Porphyromonas gingivalis* HOT-619 was significantly higher in all groups of DTC patients and that of an OTU corresponding to *Corynebacterium* species was significantly higher in all groups other than gastric cancer patients compared to that in control subjects (*P* < 0.01) (Figure 5). In tongue/pharyngeal cancer patients, OTUs corresponding to *F. nucleatum* HOT-200, *S. parasanguinis* II HOT-411, and *Neisseria* species were also more abundant (*P* < 0.01). The gastric cancer patients exhibited high relative abundance of an OTU corresponding to the *Neisseria* species and the colorectal cancer patients showed high relative abundance of an OTU corresponding to *A. odontolyticus* HOT-701 (*P* < 0.01). These results suggested that the associations between salivary microbiota and DTC differed depending on the cancer sites.

## Discussion

This case-control study of patients with various DTCs demonstrated an association of salivary microbiota with DTC. The salivary bacterial diversity was higher in DTC patients than that in control subjects. This result was consistent with other observed indices of bacterial diversity, such as Simpson index and phylogenetic diversity (Supplementary Figure S2). Particularly, tongue/pharyngeal and esophageal cancer patients demonstrated high bacterial diversity. In addition, several species-level OTUs characteristic of each DTC were identified using the LEfSe method. It was interesting that some of the OTUs were characteristic among several DTC in common, even though distances from the oral cavity to each DTC varied.

We identified the 11 bacterial species that were characteristic of DTC patients using the LEfSe method. *P. gingivalis*, a periodontal pathogen included in these 11 species, plays a key role in the development of periodontitis by invasion of epithelial cells or by interfering with the host immunity (Hajishengallis and Lamont, 2014). In this study, the relative abundance of *P. gingivalis* was significantly higher in saliva of all groups of DTC patients than that of control subjects, even though there was no difference in PPD between any groups of DTC patients and control subjects, which has been frequently associated with the presence of *P. gingivalis*. The reason for the association between higher abundance of *P. gingivalis* in saliva and DTC is unknown. However, we cannot exclude the possibility that *P. gingivalis* affects DTC incidence through an unknown pathway. In addition, *F. nucleatum*, a periodontal pathogen, was also identified as a differentially abundant OTU in cancer patients and was more abundant in tongue/pharyngeal or esophageal cancer patients. The association between periodontal pathogens in the saliva and DTC should be explored to elucidate the etiology of DTC.

We found that an OTU corresponding to *Neisseria* species was more abundant in tongue/pharyngeal, gastric, or colorectal cancer patients. *Neisseria* species are capable of producing a high amount of acetaldehyde in a medium containing ethanol *in vitro* (Muto et al., 2000; Moritani et al., 2015). In addition, the acetaldehyde level in saliva was reported to be associated with the increased risk for developing upper DTC (Salaspuro, 2003). Therefore, *Neisseria* species might be associated with DTC due to the production of acetaldehyde in the oral cavity after alcohol consumption. However, our previous study suggested that their phenotypes *in vitro* do not necessarily reflect their behavior *in vivo* because of interactions in the bacterial community (Yokoyama et al., 2018). To elucidate the acetaldehyde-mediated carcinogenesis, further comprehensive studies of these *Neisseria* species *in vitro* and *in vivo* are required.

In the present study, we conducted dental examinations for all participants and evaluated the oral health conditions, including PPD and BOP, which have seldom been evaluated in previous studies of the oral microbiota and DTC. While gingival inflammation (based on BOP) was significantly severe in tongue/pharyngeal, esophageal, or colorectal cancer patients, there was no statistical difference in PPD. This result suggests that the difference in the salivary microbiota between DTC patients and control subjects does not merely reflect subgingival bacteria shed from deep periodontal pockets with periodontitis. There may be a direct relationship between the salivary microbiota and DTC, and an indirect relationship via gingivitis. The elucidation of these etiologies would provide us with valuable information to understand the association between the oral microbiota and DTC. However, the sample size in this study was not large enough to confirm whether the differentially abundant OTUs are related to DTC independent of the gingival condition. Additionally, the small sample size did not allow us to evaluate the relationship between the oral microbiota and DTC accounting for the detailed cancer sites (e.g., gingiva, buccal mucosa, and tongue in the oral cavity or rectum and colon in the intestine) or cancer types (e.g., adenocarcinoma and squamous cell carcinoma). Further investigation with a larger sample size is needed to elucidate the relationship between the oral microbiota and DTC in detail.

We also performed LEfSe analysis between each cancer group and matched control subjects (Supplementary Figure S3). Although the sample size of each group was not large, several OTUs specific to each cancer group were detected including a subset of the 11 differentially abundant OTUs found in all DTC patients. It is particularly interesting that 5 of these 11 OTUs were detected by LEfSe analysis in colorectal cancer patients, even though colorectal cancers are physically most distant from the oral cavity, and its association with the salivary microbiota was likely to be masked by the other cancers. There might be a stronger association between the salivary microbiota and colorectal cancer than exists with the other cancers. More detailed analyses with larger sample sizes will be required to identify the respective associations of the salivary microbiota with each DTC.

## Conclusion

In conclusion, the present case-control study demonstrated that the bacterial diversity and composition of saliva is associated with DTC. Understanding of these associations can help in establishing a novel concept for cancer prediction or diagnosis based on bacterial composition. The monitoring of salivary microbiota may help predict the development of DTC and assist with health maintenance.

## Data Availability

The datasets generated for this study can be found in the DDBJ Sequence Read Archive under the accession number DRA008522.

## Ethics Statement

This study was carried out in accordance with the recommendations of the ethics committee of Kyushu University with written informed consent from all subjects. All subjects gave written informed consent in accordance with the Declaration of Helsinki. The protocol was approved by the ethics committee of Kyushu University (approval number 27–37).

## Author Contributions

SK wrote the first draft of the manuscript. SK, TT, and YY edited the manuscript. TT, MF, and YS collected the clinical data and sample from control subjects. KN, MI, MMo, MMa, and YT collected the clinical data and samples from patients. MA and RM performed the molecular analysis. SK, TT, and KT performed the bioinformatics and statistical analysis. SK, TT, YY, KT, MI, MMo, MMa, and YT contributed to the conception and design of the study. YK and TN supervised the Hisayama study. All authors read and approved the submitted version of the manuscript.

## Conflict of Interest Statement

The authors declare that the research was conducted in the absence of any commercial or financial relationships that could be construed as a potential conflict of interest.

## Supplementary Material

FIGURE S1 Mean relative abundances of bacterial genera in digestive tract cancer (DTC) patients and control subjects. Only 14 genera with a mean relative abundance of ≥1% in DTC patients or control subjects are shown. Error bars show standard error. Significant differences were determined using the Mann–Whitney *U*-test. ^*^*P* < 0.05 and ^∗∗^*P* < 0.01.

FIGURE S2 Simpson index and phylogenetic diversity of saliva in digestive tract cancer (DTC) patients, each patient group, and control subjects. Boxplots show these indices of bacterial diversity in DTC patients, each patient group, and control subjects. Phylogenetic diversity was calculated using the pd function in the picante package of R. Significant differences are determined between DTC patients and control subjects, or respectively determined between each patient group and control subjects using the Mann–Whitney *U*-test. ^*^*P* < 0.05 and ^∗∗^*P* < 0.01.

FIGURE S3 Bacterial species corresponding to the differentially abundant operational taxonomic units (OTUs) between each patient group and matched control subjects. Bar plots show linear discriminant analysis (LDA) scores of each OTU. The LDA score indicates the effect size of each OTU and OTUs with an LDA score >3.0 are shown. The differentially abundant OTUs in patients and control subjects are depicted using different colors. Oral taxon identifications are in parentheses following bacterial names.
