# Supplementary figures and images for: Characteristics of the Salivary Microbiota in Patients With Various Digestive Tract Cancers
Source: Front Microbiol. 2019 Aug 2;10:1780. doi: 10.3389/fmicb.2019.01780 (PMC6688131; doi:10.3389/fmicb.2019.01780)

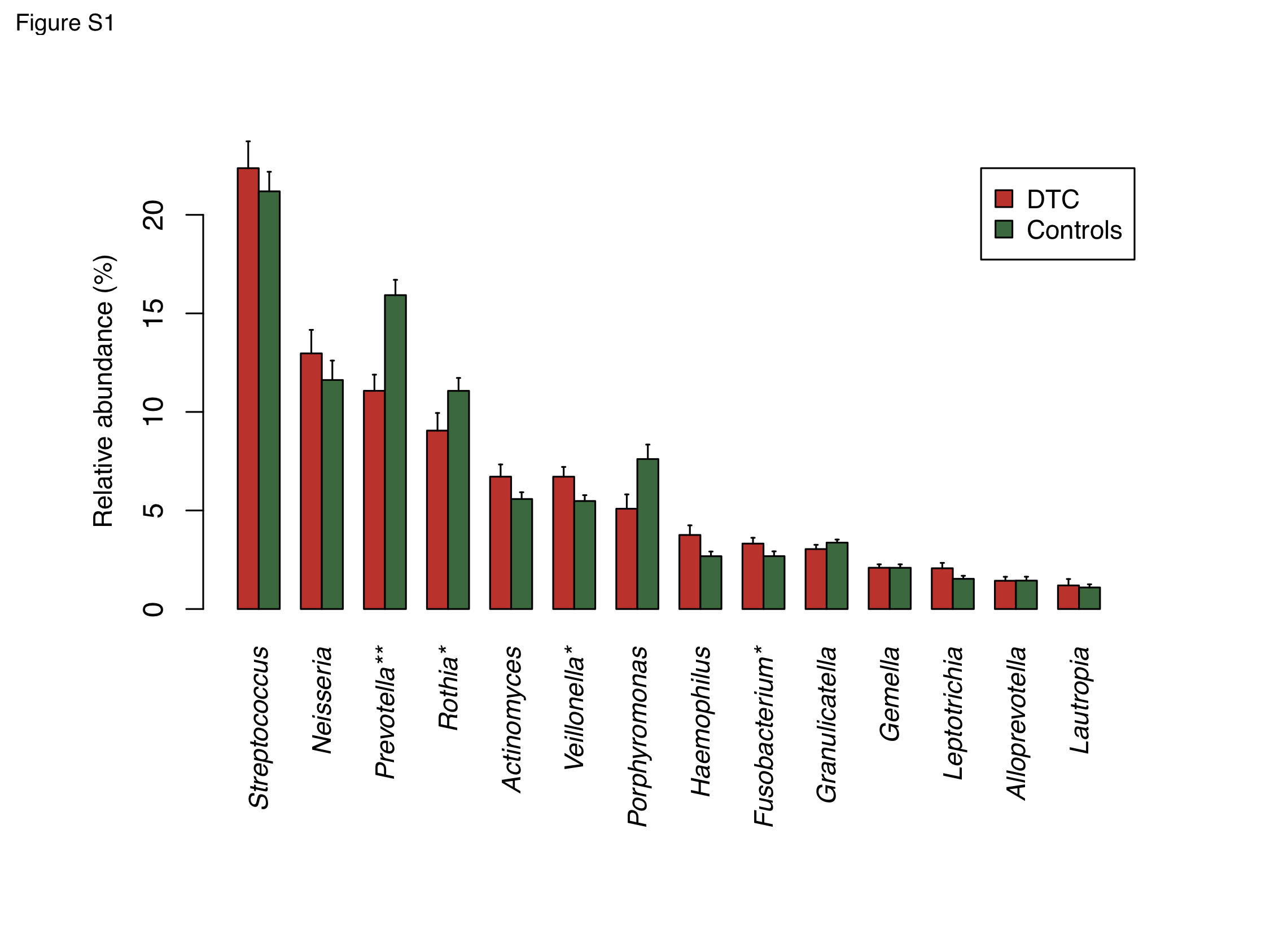

Supplement: FIGURE S1 — Mean relative abundances of bacterial genera in digestive tract cancer (DTC) patients and control subjects. Only 14 genera with a mean relative abundance of ≥1% in DTC patients or control subjects are shown. Error bars show standard error. Significant differences were determined using the Mann–Whitney U-test. *P < 0.05 and ∗∗P < 0.01. [file Image_1.TIFF]

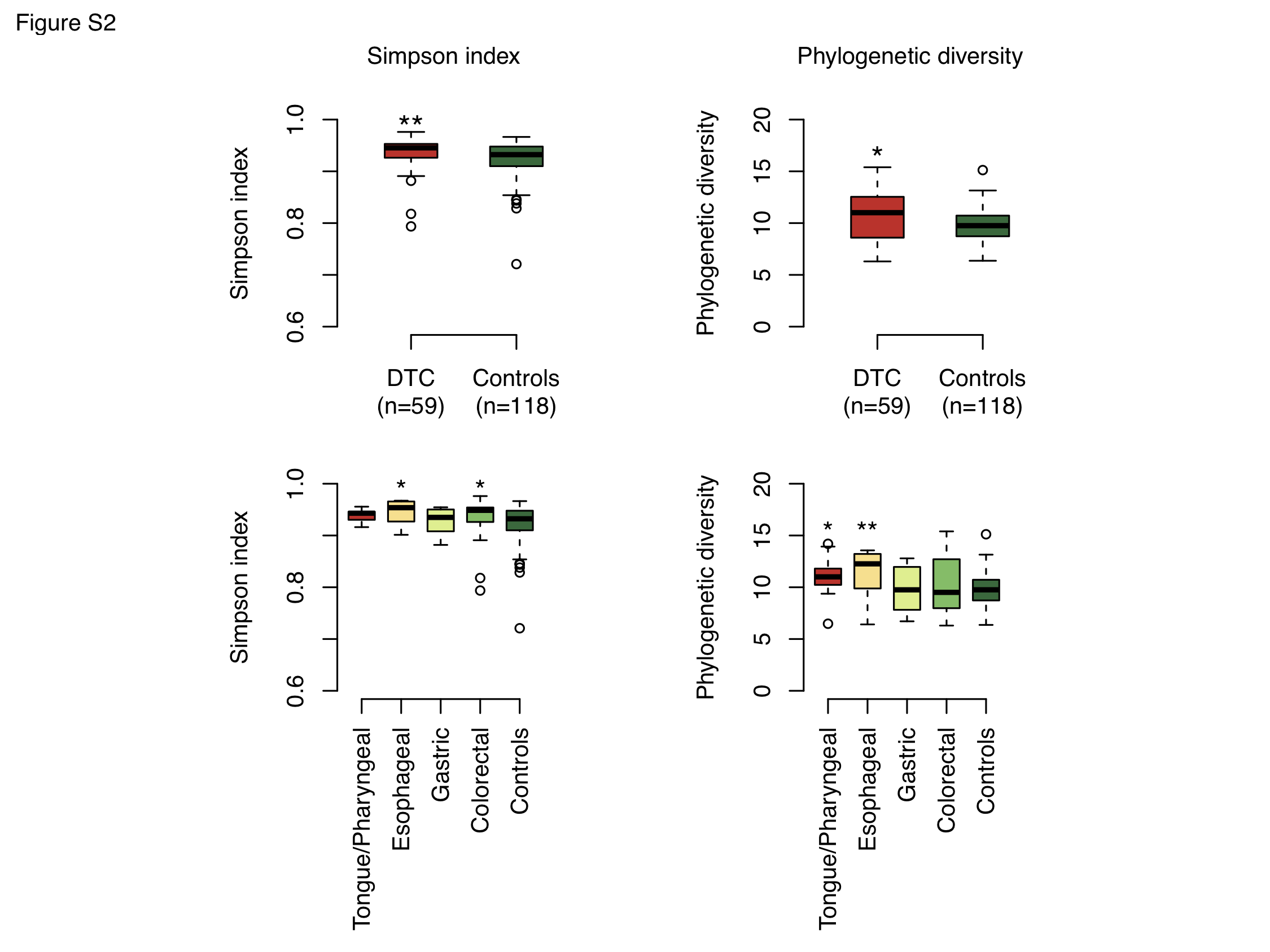

Supplement: FIGURE S2 — Simpson index and phylogenetic diversity of saliva in digestive tract cancer (DTC) patients, each patient group, and control subjects. Boxplots show these indices of bacterial diversity in DTC patients, each patient group, and control subjects. Phylogenetic diversity was calculated using the pd function in the picante package of R. Significant differences are determined between DTC patients and control subjects, or respectively determined between each patient group and control subjects using the Mann–Whitney U-test. *P < 0.05 and ∗∗P < 0.01. [file Image_2.TIFF]

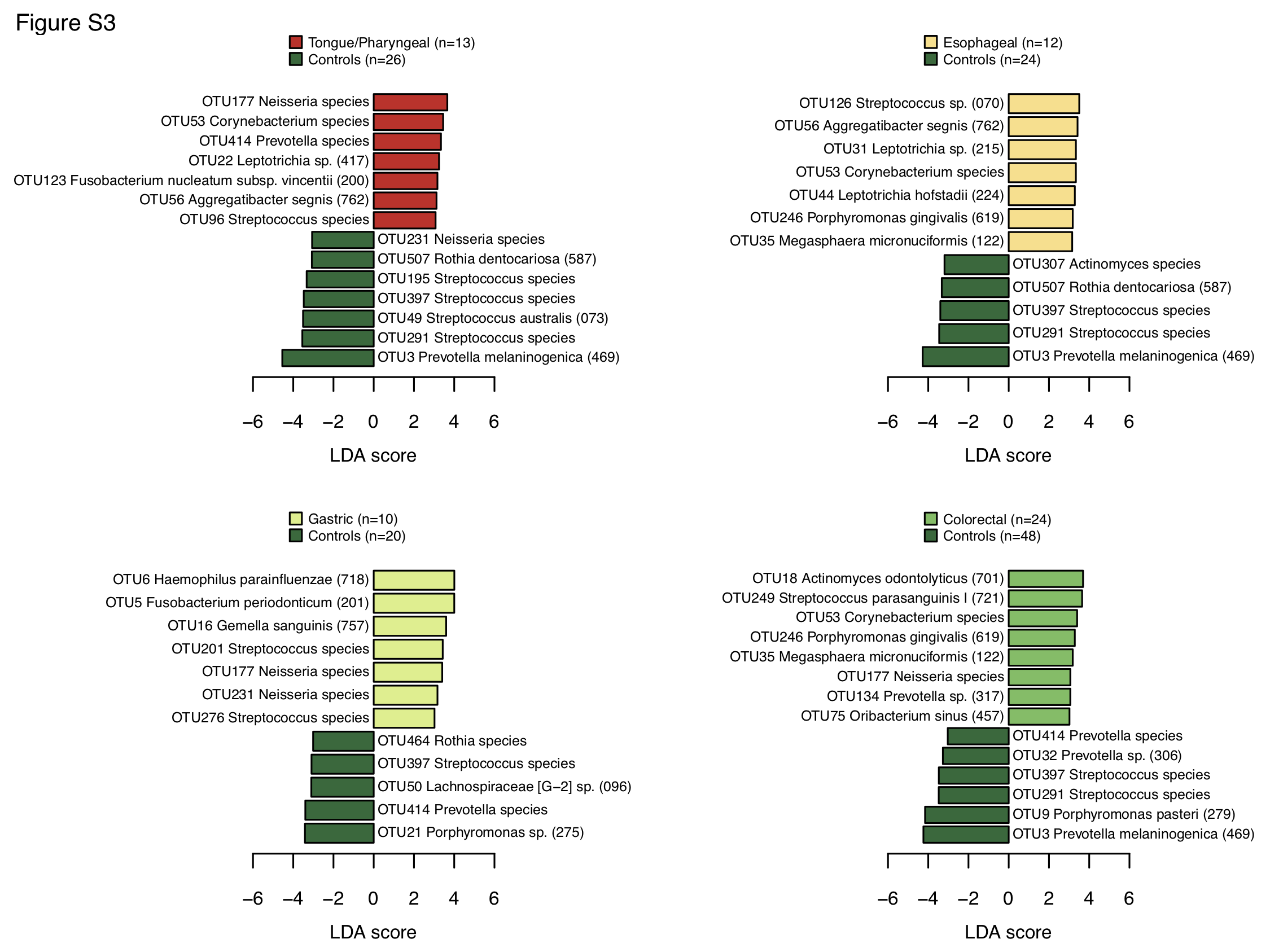

Supplement: FIGURE S3 — Bacterial species corresponding to the differentially abundant operational taxonomic units (OTUs) between each patient group and matched control subjects. Bar plots show linear discriminant analysis (LDA) scores of each OTU. The LDA score indicates the effect size of each OTU and OTUs with an LDA score >3.0 are shown. The differentially abundant OTUs in patients and control subjects are depicted using different colors. Oral taxon identifications are in parentheses following bacterial names. [file Image_3.TIFF]
